# Clinical Outcomes of Single-incision Laparoscopic Appendectomy Versus Conventional Laparoscopic Appendectomy in Adult Acute Appendicitis

**DOI:** 10.14789/ejmj.JMJ24-0032-OA

**Published:** 2024-12-31

**Authors:** SHINTARO KOHAMA, KUNIHIKO NAGAKARI, MASAKAZU OHUCHI, KAZUHIRO TAKEHARA, KUMPEI HONJO, SHUN ISHIYAMA, KIICHI SUGIMOTO, SHINICHI OKA, JIRO YOSHIMOTO, MASAKI FUKUNAGA, YOICHI ISHIZAKI, KAZUHIRO SAKAMOTO

**Affiliations:** 1 Department of Digestive Surgery, Juntendo University Urayasu Hospital, Chiba, Japan; 1 Department of Digestive Surgery, Juntendo University Urayasu Hospital, Chiba, Japan; 2 Department of Coloproctological Surgery, Graduate School of Medicine, Juntendo University, Tokyo, Japan; 2 Department of Coloproctological Surgery, Graduate School of Medicine, Juntendo University, Tokyo, Japan

**Keywords:** single-incision laparoscopic appendectomy, laparoscopic appendectomy, propensity score matching

## Abstract

**Objectives:**

Laparoscopic surgery is widely performed for acute appendicitis. We started conventional 3-port laparoscopic appendectomy (CLA) in 1995 and introduced single-incision laparoscopic appendectomy (SILA) in 2009. This study compared perioperative outcomes between SILA and CLA to evaluate the usefulness of SILA.

**Design:**

Retrospective observational study.

**Methods:**

The study included 568 patients who underwent emergency or semi-emergency surgery for acute appendicitis (327 by CLA and 241 by SILA) at our hospital between January 2009 and December 2020. Perioperative outcomes were compared between SILA and CLA after adjusting for patient demographics by propensity score matching (PSM).

**Results:**

PSM gave a matched sample of 224 patients in each of the CLA and SILA groups. There were significant differences between the two groups in time to initiation of oral intake, frequency of postoperative analgesic use, and length of postoperative hospital stay. Time to oral intake was significantly shorter in the SILA group (p = 0.02). Frequency of use of all analgesics, flurbiprofen axetil, and loxoprofen sodium was significantly higher in the SILA group (p < 0.01, p = 0.04, p < 0.01, respectively). The length of postoperative hospital stay was significantly shorter in the SILA group (p < 0.01). The incidence of postoperative complications did not differ significantly between the two groups.

**Conclusions:**

Although SILA required significantly more postoperative analgesics than CLA, pain could be controlled by oral analgesics, and patients could be discharged earlier. Postoperative complications were comparable between the two groups. SILA was a safe and feasible procedure for adult acute appendicitis.

## Introduction

Acute appendicitis is one of the most frequent abdominal emergencies, often presenting with right lower quadrant abdominal or epigastric pain. Laparoscopic appendectomy (LA) was first reported by Semm et al. in 1980^1)^. Since then, its indications have expanded to perforated appendicitis and abscess-forming appendicitis^2)^. Single-incision laparoscopic appendectomy (SILA) has also been introduced recently in an attempt to improve esthetic outcomes and reduce surgical invasion. Our hospital introduced conventional laparoscopic appendectomy (CLA) in 1995^3)^ and SILA in 2009.

Overseas meta-analyses which compared SILA with CLA^4-7)^ have shown that although SILA is associated with increased operative time, both techniques are comparable in terms of postoperative complications and length of hospital stay. Some reports have described increased postoperative pain in SILA^4)^, while others have indicated that pain is equivalent between the two techniques^5, 6)^, showing a lack of consensus on this issue. In Japan, Moriguchi et al. reported a randomized controlled trial (RCT) of SILA versus CLA in pediatric appendicitis^8)^, but no comparative study in adults has been reported.

In this study, we compared perioperative outcomes of SILA with those of CLA using propensity score matching (PSM) in order to evaluate the usefulness of SILA in adult acute appendicitis.

## Materials and Methods

The study included 568 patients who underwent emergency or semi-emergency surgery for acute appendicitis (327 by CLA and 241 by SILA) at our hospital between January 2009 and December 2020. The surgical indication for acute appendicitis was based on a comprehensive evaluation of the patient’s medical history, abdominal findings, blood tests, and computed tomography, and appendicitis phlegmonosa or more severe appendicitis was considered to be eligible for surgery.

Patients were indicated for SILA if they did not have generalized peritonitis, extensive abscess formation on computed tomography, or significant adhesions in the peritoneal cavity. For cases meeting these criteria, the surgical approach was selected on a case-by-case basis, depending on the surgeon’s skill and judgment.

Patients undergoing elective surgery and those with pathologically confirmed malignancy were excluded. Patients undergoing double-incision laparoscopic appendectomy were also excluded, except for cases in which the procedure was started with SILA and then a suprapubic port was added, which were classified as additional port cases and included in the SILA group.

**Figure 1:**
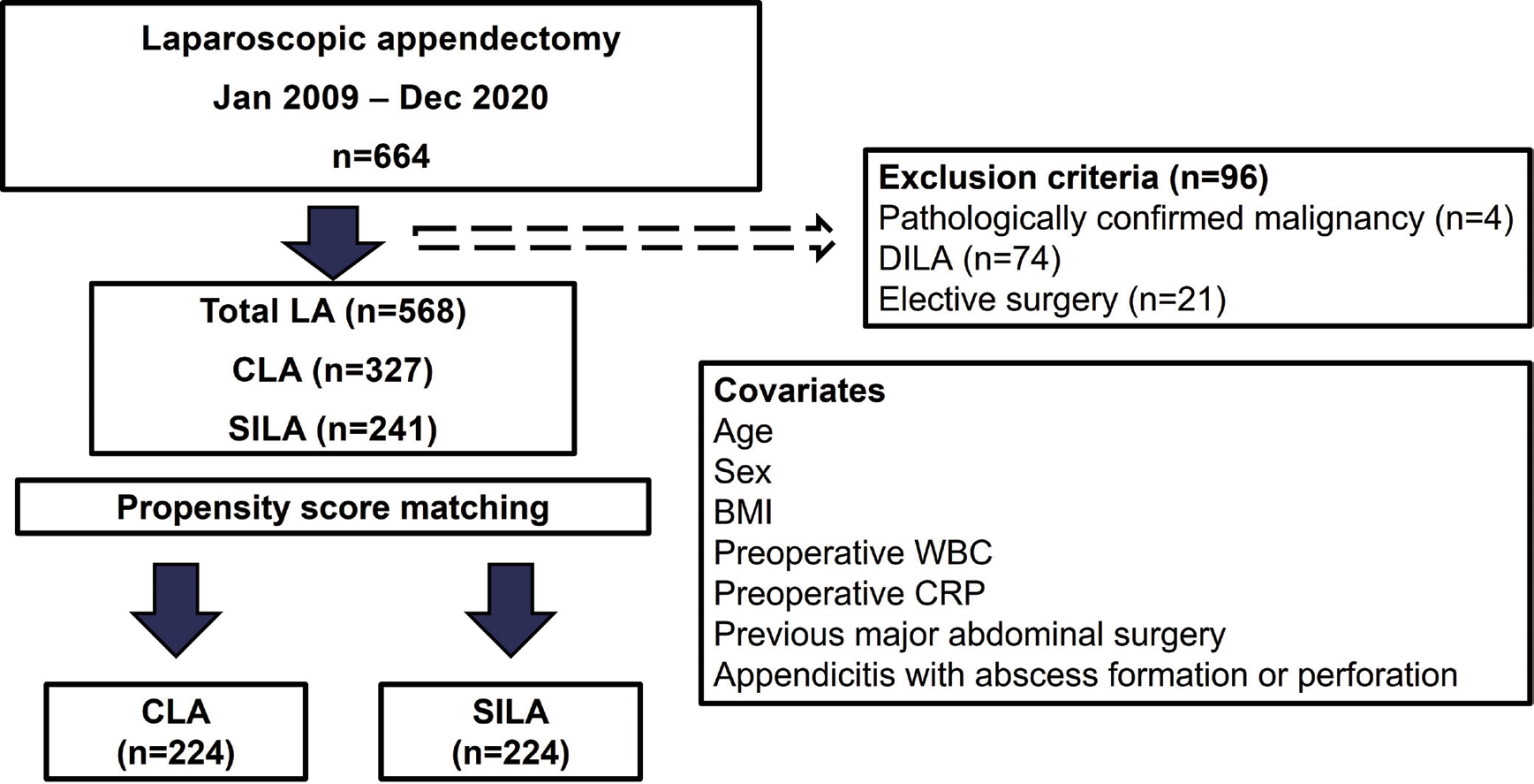
Flowchart of the study CLA, conventional laparoscopic appendectomy; CRP, C-reactive protein; DILA, double-incision laparoscopic appendectomy; LA, laparoscopic appendectomy; SILA, single-incision laparoscopic appendectomy; WBC, white blood cell count.

The demographic and clinical characteristics of patients in the CLA and SILA groups were adjusted using PSM (age, sex, body mass index, preoperative white blood cell count [WBC], preoperative C-reactive protein [CRP], prior surgery, abscess formation, and perforation). The matched groups were compared for perioperative outcomes (operative time, blood loss, additional port insertion, time to oral intake, frequency of postoperative analgesic use, postoperative complications, postoperative hospital stay, and pathological findings; Figure 1). All grades of postoperative complications were recording using the Clavien-Dindo classification^9)^.

Oral intake was resumed the day after surgery, provided that there were no signs of paralytic ileus, such as vomiting or abdominal distension. Although no clinical pathway was established for appendectomy, the decision for discharge was made comprehensively by the surgeon, after confirming that postoperative pain was adequately managed with oral analgesics and that there was no worsening of abdominal symptoms or inflammatory markers following oral intake.

The postoperative analgesic use protocol was as follows. Acetaminophen or flurbiprofen axetil was used immediately after surgery, followed by pentazocine. Loxoprofen sodium was used after the patient started drinking water. Loxoprofen sodium was used as needed, up to 3 doses a day. The number of doses of each analgesic was counted as the frequency of analgesic use.

This study was carried out in accordance with the principles embodied in the World Medical Association’s Declaration of Helsinki, and was approved by the Juntendo University School of Medicine Ethics Committee for Medical Research (E24-0010-U01). The requirement for informed consent was waived owing to the retrospective and observational design of the study. An opt-out approach was used by providing access to a written disclosure on the study website (URL: https://www.gcprec.juntendo.ac.jp/kenkyu/detail/6503).

### Surgical procedure

The operation was performed under general anesthesia in the supine position. Cefmetazole sodium, a second-generation cephem antibiotic with a spectrum covering anaerobic bacteria, was administered as prophylaxis against infection immediately before the operation. The umbilicus was completely inverted and cleaned with chlorhexidine gluconate solution. Both the surgeon and the scopist stood on the patient’s left side, and the monitor was positioned caudally and to the right of the patient.

**Figure 2:**
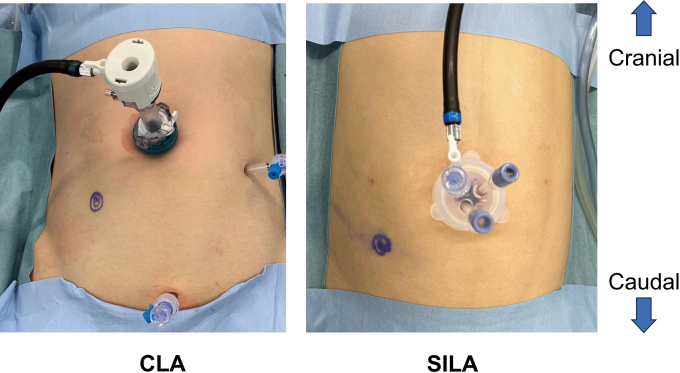
Port placement CLA, conventional laparoscopic appendectomy; SILA, single-incision laparoscopic appendectomy.

In CLA, a skin incision of approximately 10 mm was placed at the umbilicus to identify the preperitoneal fat and reach the peritoneal cavity. A 12-mm port was placed, and under insufflation at 10 mmHg and observation of the peritoneal cavity, two 5-mm ports were placed in the left lower abdomen and suprapubic area. For SILA, a skin incision of approximately 15 mm was made to fit within the umbilical ring based on the patient’s umbilical shape, and a small craniocaudal fascial incision centered at the umbilicus was performed. Although the incision was designed to fit within the umbilical ring, the exact wound length was not routinely measured for each case. Three 5-mm ports were placed in an inverted triangle using the SILS Port 5 mm^TM^ system (Medtronic, Dublin, Ireland) or E-Z Access Mini Mini Type^TM^ system (Hakko Medical, Nagano, Japan) (Figure 2). In SILA, the ENDOEYE FLEX Tip Curved Videoscope^TM^ or the ENDOEYE Rigid Videoscope^TM^ (Olympus Medical Systems, Tokyo, Japan) was used to improve the visibility of the device tip. There were no restrictions on the scopes used for CLA.

**Figure 3:**
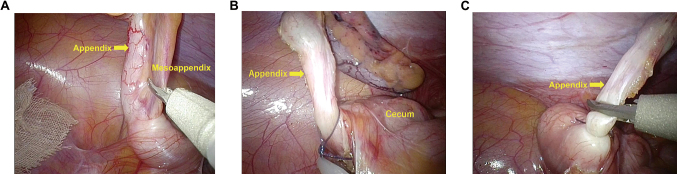
Laparoscopic views during single-incision laparoscopic appendectomy (A) Dissection and coagulation of the mesoappendix. (B) Ligation of the base of the appendix. (C) Cutting of the appendix.

The patient was repositioned to the Trendelenburg position with left tilt. After identification of the appendix, the mesoappendix was dissected with laparoscopic coagulation shears toward the root (Figure 3A). The appendicular root was ligated with a single loop of 2-0 or 0 Surgitie™ (Medtronic, Dublin, Ireland; Figure 3B), and the appendix was removed with the laparoscopic coagulation shears (Figure 3C). Sufficient suction was performed in cases of abscess formation or peritonitis. The removed appendix was collected in a specimen collection bag in CLA, whereas in SILA, no collection bag was used because the wound margin was covered by a protector. Umbilical closure was achieved by closing the fascial incision with 2-0 monofilament absorbable suture and then closing the subcutaneous layer of all port wounds with 4-0 monofilament absorbable suture.

### Statistical analysis

In comparing the CLA and SILA groups, PSM was used to adjust for confounding factors. Continuous variables were analyzed with the Mann-Whitney U test, and categorical variables with the chi-square test. Differences were considered statistically significant if the P-value was less than 0.05. All statistical analyses were performed using SPSS ver. 29.0 (IBM Corp., Armonk, NY, USA).

## Results

### Patient characteristics

The baseline demographic and clinical characteristics of the 327 patients in the CLA group and 241 patients in the SILA group who underwent each procedure at our hospital between January 2009 and December 2020 are shown in Table 1. A pre- matching comparison showed that the SILA group was younger (p < 0.01), had lower preoperative inflammatory response (p = 0.04 for WBC, p < 0.01 for CRP), had fewer prior surgeries (p = 0.01), and was less likely to have perforated appendicitis or intraperitoneal abscess formation (p < 0.01). Adjustment for patient demographics using PSM resulted in a matched sample of 224 patients from the CLA group and 224 patients from the SILA group (Figure 1). After PSM, there were no significant differences in patient demographics between the two groups (Table 2).

**Table 1:** Patient characteristics (overall)

	CLA(n = 327)	SILA(n = 241)	P-value
Age(yr)	40 ± 1	35 ± 1	< 0.01*
Sex(male/female)	184/143	130/111	0.58
BMI(kg/m^2^)	21.9 ± 0.2	21.7 ± 0.2	0.49
Preoperative WBC(/μl)	13,372 ± 236	12,412 ± 257	0.04*
Preoperative CRP(mg/dl)	7.4 ± 0.4	2.9 ± 0.2	< 0.01*
Prior surgery(+/-)	17(5%)/310(95%)	3(1%)/238(99%)	0.01*
Abscess formation(+/-)	43(13%)/284(87%)	12(5%)/229(95%)	< 0.01*
Perforation(+/-)	32(10%)/295(90%)	5(2%)/236(98%)	< 0.01*

Values are presented as mean ± standard error of the mean or number(%). An asterisk indicates a significant difference(p < 0.05). BMI, body mass index; CLA, conventional laparoscopic appendectomy; CRP, C-reactive protein; SILA, single-incision laparoscopic appendectomy; WBC, white blood cell count.

**Table 2:** Patient characteristics (after matching)

	CLA(n = 224)	SILA(n = 224)	P-value
Age(yr)	36 ± 1	35 ± 1	0.48
Sex(male/female)	127/97	123/101	0.7
BMI(kg/m^2^)	21.6 ± 0.3	21.7 ± 0.2	0.96
Preoperative WBC(/μl)	13,197 ± 288	12,592 ± 260	0.36
Preoperative CRP(mg/dl)	4.0 ± 0.3	3.0 ± 0.3	0.13
Prior surgery(+/-)	3(1%)/221(99%)	3(1%)/221(99%)	1
Abscess formation(+/-)	16(7%)/208(93%)	12(5%)/212(95%)	0.43
Perforation(+/-)	11(5%)/213(95%)	5(2%)/219(98%)	0.13

Values are presented as mean ± standard error of the mean or number(%). There were no significant differences in patient characteristics after propensity score matching. BMI, body mass index; CLA, conventional laparoscopic appendectomy; CRP, C-reactive protein; SILA, single-incision laparoscopic appendectomy; WBC, white blood cell count.

### Perioperative outcomes

Perioperative outcomes were compared between the CLA and SILA groups after adjusting for patient demographics (Tables 3 and 4). No significant differences were found in operative time or blood loss between the two groups. An additional 5-mm port was placed in 3 patients (1.3%) in the CLA group and 5 patients (2.2%) in the SILA group due to technical difficulties caused by severe inflammation or abscess formation. These additional ports were placed in the right lower abdomen in CLA and in the suprapubic area in SILA, and were used for drain placement. There was no significant difference in the additional port insertion rate between the two groups.

**Table 3:** Operative results

	CLA(n = 224)	SILA(n = 224)	P-value
Operative time(min)	67 ± 2	60 ± 2	0.08
Blood loss(ml)	8 ± 1	5 ± 0.4	0.19
Additional port insertion	3(1.3%)	5(2.2%)	0.48
Time to oral intake(day)	1.6 ± 0.1	1.5 ± 0.1	0.02*
Overall frequency of analgesic use	3.0 ± 0.2	3.8 ± 0.2	< 0.01*
Wound infection	4(1.8%)	2(0.9%)	0.41
Paralytic ileus	3(1.3%)	1(0.5%)	0.32
Postoperative intraperitoneal abscess	1(0.5%)	5(2.2%)	0.1
Postoperative hospital stay(day)	7.9 ± 0.3	6.5 ± 0.2	< 0.01*
Pathological findings (catarrhal/phlegmonous/gangrenous)	10/90/124	9/80/135	0.57

Values are presented as mean ± standard error of the mean or number(%). An asterisk indicates a significant difference(p < 0.05). CLA, conventional laparoscopic appendectomy; SILA, single-incision laparoscopic appendectomy.

**Table 4:** Frequency of postoperative analgesic use by drug type

	CLA(n = 224)	SILA(n = 224)	P-value
All analgesics	3.0 ± 0.2	3.8 ± 0.2	< 0.01*
Acetaminophen	0.3 ± 0.1	0.5 ± 0.1	0.39
Flurbiprofen axetil	1.3 ± 0.1	1.4 ± 0.1	0.04*
Pentazocine	0.2 ± 0.1	0.3 ± 0.1	0.18
Loxoprofen sodium	1.1 ± 0.2	1.6 ± 0.1	< 0.01*

Values are presented as mean ± standard error of the mean. An asterisk indicates a significant difference(p < 0.05). CLA, conventional laparoscopic appendectomy; SILA, single incision laparoscopic appendectomy.

The variables that differed significantly between the two groups were time to initiation of oral intake, frequency of analgesic use, and length of postoperative hospital stay. Time to oral intake was significantly shorter in the SILA group (p = 0.02). Frequency of use of all analgesics, flurbiprofen axetil, and loxoprofen sodium was significantly higher in the SILA group (p < 0.01, p = 0.04, p < 0.01, respectively). The frequency of pentazocine use was not significantly different between the two groups. The length of postoperative hospital stay was significantly shorter in the SILA group (p < 0.01).

Postoperative complications included Clavien-Dindo Grade I wound infection (all affecting the umbilical wound), Grade I paralytic ileus, and Grade II postoperative intraperitoneal abscess. The incidence of these postoperative complications did not differ significantly between the two groups. A review of late complications showed no abdominal incisional hernia in either group.

**Figure 4:**
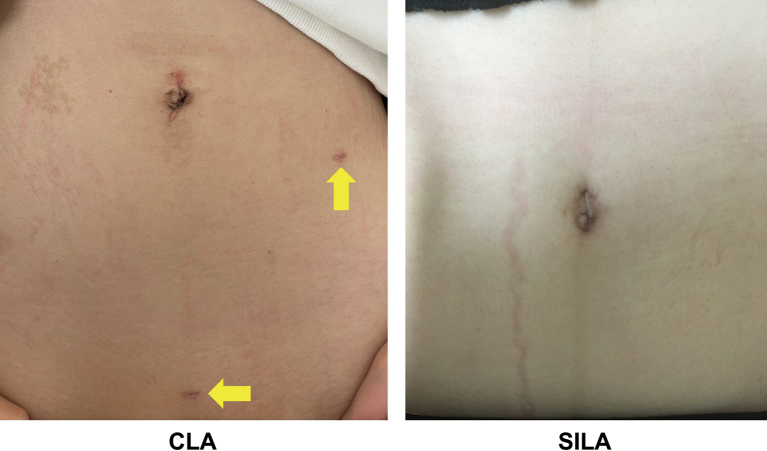
Postoperative scar (1-month after surgery) The yellow arrows indicate the 5-mm port scars from CLA. The SILA scar is contained within the umbilical ring and is inconspicuous. CLA, conventional laparoscopic appendectomy; SILA, single-incision laparoscopic appendectomy.

The 1-month postoperative scar of the CLA and SILA is shown in Figure 4. The SILA scar was inconspicuous, as it was confined within the umbilical ring.

## Discussion

Since Dr. Kurt Semm reported the first LA case in 1980^1)^, LA has been widely performed. With concurrent advances in laparoscopic techniques and equipment, LA has now become the standard procedure for acute appendicitis^10)^. LA has been shown to be superior to open surgery in terms of time to initiation of oral intake, incidence of wound infection, and length of postoperative hospital stay, regardless of the severity of appendicitis^2, 3)^.

The SAGES guidelines^11)^ state as follows: “Laparoscopic appendectomy is a safe and effective method for the treatment of uncomplicated appendicitis and may be used as an alternative to standard open appendectomy (level I, grade A)…Laparoscopic appendectomy may be performed safely in patients with perforated appendicitis (level II, grade B) and is possibly the preferred approach (level III, grade C).” The Japanese guidelines for endoscopic surgery^12)^ also state as follows: “Laparoscopic surgery for acute appendicitis is recommended because it reduces the incidence of postoperative complications such as wound infection and paralytic ileus, despite the increased operative time compared to open surgery.”

Although LA is usually performed through three ports, Pelosi et al. reported SILA in 1992 as a surgical technique requiring fewer ports^13)^, and it subsequently came into widespread use.

According to the WSES Jerusalem guidelines^14)^, “Single-incision laparoscopic appendectomy is basically feasible, safe, and as effective as conventional three-port laparoscopic appendectomy, operative times are longer, requires higher doses of analgesia, and is associated with a higher incidence of wound infection.” Overseas meta-analyses^4-7)^ showed that SILA was associated with an increased operative time, but was comparable to CLA in terms of postoperative complications and length of hospital stay. In regard to postoperative pain, some reports suggest increased pain with SILA^4)^, while others indicate that pain is equivalent between the two techniques^5, 6)^. An RCT reported by Kyeong et al. showed a significantly shorter operative time and significantly less postoperative pain with SILA^15)^. A PSM analysis by Sung et al. showed that operative time tended to be shorter with SILA and postoperative pain was comparable between the two techniques^16)^, while Won et al. reported similar operative time and postoperative pain, but significantly shorter hospital stays with SILA^17)^.

In Japan, Moriguchi et al. conducted an RCT of 72 cases of pediatric appendicitis^8)^. This study compared SILA and CLA for pediatric appendicitis performed by attending pediatric surgeons and surgeons in training. The authors concluded that both techniques were safe, with no significant differences in perioperative outcomes between them when performed by surgeons with similar levels of experience.

Given that there were no previous Japanese studies comparing CLA and SILA for appendicitis in adults, the present study compared the perioperative outcomes of CLA and SILA using PSM in order to evaluate the usefulness of SILA in these patients.

No significant difference in operative time was found between the two groups. This was consistent with the previous reports^16, 17)^. Most overseas meta- analyses showed a longer operative time with SILA than with CLA^4-7)^, with the exception of Kyeong et al., who reported that operative time was shorter with SILA than with CLA^15)^. They attributed this to SILA using a natural orifice as the port insertion site, thus shortening the time required for port insertion compared with CLA, and to specimens being removed directly through the incisional wound, thus eliminating the need for a collection bag.

Time to initiation of oral intake and length of postoperative hospital stay were significantly shorter in the SILA group. Since the decision to initiate oral intake and discharge was made at the discretion of the surgeon, it is possible that unadjusted bias, such as earlier discharge recommended by the surgeon in SILA cases, may have contributed to this result.

With regard to postoperative analgesics, the frequency of all analgesic use was significantly higher with SILA. The most commonly used analgesics were loxoprofen sodium in the SILA group and flurbiprofen axetil in the CLA group. These nonsteroidal anti-inflammatory drugs (NSAIDs) were used significantly more frequently in the SILA group than in the CLA group. The least used analgesic was pentazocine in both groups, with a similar frequency of use in both groups. Intravenous analgesics were used on the day of surgery and these were switched to oral analgesics the day after surgery, when patients began drinking water. The results showed that NSAIDs were used more frequently in the SILA group both on the day of surgery and the day after surgery and beyond. On the other hand, the length of hospital stay was significantly shorter with SILA. Although SILA was associated with more frequent use of analgesics, the fact that pain could be controlled with oral analgesics may have contributed to earlier discharge from the hospital. Postoperative pain is generally caused by injury to the muscles and the parietal peritoneum. Ahn et al. have suggested that the length of the fascial incision is closely related to postoperative wound pain^18)^. They noted that although the skin incision in the umbilical area is small, the actual length of the fascial incision is longer in SILA compared to CLA, and the use of multiple laparoscopic instruments through a small incision window can irritate the wound. Considering these findings, we speculate that the higher need for analgesics in SILA compared to CLA may be attributed to the procedural differences between the two techniques. In CLA, the umbilical fossa is enlarged to accommodate a 12-mm port without making a fascial incision, which may result in minimal trauma to the fascia. In contrast, SILA requires a craniocaudal fascial incision around the umbilical fossa, resulting in a longer fascial incision. Moreover, operating both the scope and forceps through a single umbilical incision may increase tissue strain and compression due to instrument interference. This can lead to greater irritation and stretching of the incision site, potentially causing more intense postoperative pain and a higher need for analgesics compared to CLA. To mitigate the increased analgesic requirement in SILA, techniques such as performing a rectus sheath block during surgery or administering stronger analgesics like pentazocine from the outset could be considered, balancing their efficacy with the potential risks of dependence and side effects. Additionally, Ahn et al. have reported that wound infiltration with 0.5% bupivacaine just before incision provided effective early postoperative pain relief^18)^. Implementing such measures could help reduce the higher analgesic demand observed in SILA patients.

There was no significant difference between the two groups in terms of postoperative complications, including wound infection. These results were consistent with overseas meta-analyses^4-7)^ and support the safety of our surgical procedure.

There are several limitations with this study. First, it was a single-center retrospective study. Second, postoperative pain was not numerically quantified using a pain scoring system, such as a visual analog scale. Another limitation is that the study did not analyze esthetic outcomes. Kyeong et al. reported that SILA is superior to CLA in terms of esthetic outcomes, although there was no difference in patient satisfaction between the two techniques^15)^. Further study is needed to demonstrate the superiority of SILA in terms of esthetic outcomes and patient satisfaction, which would then show the increased usefulness of the technique.

Although SILA required significantly more postoperative analgesics than CLA, pain could be controlled by oral analgesics, and patient could be discharged earlier. Postoperative complications were comparable between the two groups. SILA was a safe and feasible procedure for adult acute appendicitis.

## Funding

The authors received no financial support for the research.

## Author contributions

SK contributed to the study concept and design; data acquisition and interpretation; statistical analysis; and drafting of the manuscript. KN, MF, YI, and KSa contributed significantly to the design of the study; the collection, analysis, and interpretation of the data; and the writing of the manuscript. MO, KT, KH, SO, and JY were involved in patient treatment and data collection. SI, KSu contributed to the manuscript revision. KSa supervised the study. All authors read and approved the final manuscript.

## Conflicts of interest statement

The authors declare that there are no conflicts of interest.
